# EEG Derived Neuronal Dynamics during Meditation: Progress and Challenges

**DOI:** 10.1155/2015/614723

**Published:** 2015-12-06

**Authors:** Chamandeep Kaur, Preeti Singh

**Affiliations:** Panjab University, Chandigarh 160036, India

## Abstract

Meditation advances positivity but how these behavioral and psychological changes are brought can be explained by understanding neurophysiological effects of meditation. In this paper, a broad spectrum of neural mechanics under a variety of meditation styles has been reviewed. The overall aim of this study is to review existing scientific studies and future challenges on meditation effects based on changing EEG brainwave patterns. Albeit the existing researches evidenced the hold for efficacy of meditation in relieving anxiety and depression and producing psychological well-being, more rigorous studies are required with better design, considering client variables like personality characteristics to avoid negative effects, randomized controlled trials, and large sample sizes. A bigger number of clinical trials that concentrate on the use of meditation are required. Also, the controversial subject of epileptiform EEG changes and other adverse effects during meditation has been raised.

## 1. Introduction

Meditation is a broad variety of practices. Meditation includes relaxation supporting techniques and various movements to achieve regulation and nurturing of well-being. It is defined as the natural process of manipulating one's state of mind and self-regulating attention level intentionally, although it is lacking a clear operational definition [1, 2]. Experienced meditators show stronger regulated features. Analysis of various meditation states has been done to explore difficult neural processes. The large and complex neuronal structures turn to more relaxed structures during meditation [3]. So meditation practitioners show distinct EEG recording patterns. In the beginning of each meditation style, Lutz et al. have categorized meditation as Focused Attention (FA) and open monitoring (OM). Travis represented another category as automatic self-transcending according to brain pattern based EEG bands. These categories define a number of meditation practices [4, 5]. Minimum efforts are required to reach the state of FA and a steady focus is achieved which is evident from less activation in the amygdala [1].

*Physiological and Neural Correlates of Meditation Therapy*. Meditation practices can change the way of thinking. Metacognitive reasoning could be made by people to predict the discrepancy between what should be thought and what is being thought. In [6], various neurophysiologic changes on cognition, hormonal, and autonomic systems have been theorized while meditating. Evidences reveal the positive effects of meditation as it increases the level of monoamines, parasympathetic activity, and gray matter density of brain regions (reflecting emotion regulation) and reduces oxidative effects. These effects result in reversal of stress mediated depression. Further research needs to be done in exploring these changes with large samples in consideration. The spectral power and coherence of EEG define delta, theta, and alpha frequency bands to characterize different meditation states.

Numerous researches have been carried out to scientifically explore the principle brain neurological changes during meditation. But neuroelectrophysiology of meditation based states is still an open question. A very small number of clinical applications of meditation have been identified and those too are lacking in control group and concurrent antidepressant medication group along with shorter follow-up period [6, 7]. Albeit some progress has been made in theorizing the neurophysiological effects under meditation [4, 8–10], evoked potential, and event related potential research in meditation [11], still rationale to quantitatively represent the neural effects is not clear.

This review is a noteworthy contribution to review existing scientific studies and future challenges on meditation effects based on changing EEG brainwave patterns. A debate on the EEG changes during meditation, controversial adverse effects of meditation, and signal processing challenges with future direction has been given below.

## 2. EEG during Meditation

A detailed quantitative analysis of neural effects under the effect of various meditation states has been discussed below. Other studies on the EEG effects of meditation could be found in Table 1.

### 2.1. Buddhist Meditation

Based on open monitoring, Buddhist meditators are characterized by high amplitude gamma oscillations [1]. A study about the impact of Buddhist meditation on emotional processing was presented [12]. There was no difference in psychological testing in experimental and control group (CG). Also, randomized design should be incorporated in the study design.

#### 2.1.1. Zen Meditation

Zen meditation, a type of Buddhist meditation, is characterized by increase in slow alpha power, reflecting more internalized attention, and fast theta power, reflecting enhanced mindfulness in frontal area [9, 13]. Long-term practitioners show alpha1 rise in frontal region and beta rise over occipital regions [14].

*Investigations on FmTheta*. During a mental effort, EEG shows a decrease in alpha and this strength goes on decreasing as the task becomes more tedious. However, frontal midline theta (FmTheta) increases during mental task. FmTheta involves attention levels that appear during mental task and meditation. It is an indicator of more demonstrative, more active, and less phobic activities [15]. It is defined on EEG as distinctive theta activity in frontal midline area. A study was performed on theta signal during concentrative task (meditation) and consecutive mental task [16]. It refers to attention maintenance during mental effort. Enhanced theta and constant occipital alpha were observed between FmTheta conditions and control. Further investigations on FmTheta can add to the underlying mechanisms of mind and body.

#### 2.1.2. CHAN Meditation

Researchers correlated occipital alpha wave rise during eye closed relaxation and frontal alpha wave (representing mindfulness) during CHAN meditation. A method explored spatial temporal properties of different alpha maps using microstate analysis in [17]. It reflected more inward attention than the CG. Another study showed that better cortical interactions are possessed during CHAN meditation and Chakra focusing practice than the CG [18]. In this study, interactions among various brain networks under alpha oscillations have been explored because of Continuous Time Wavelet Transform. This nonlinear independence analysis identified the dominant alpha epochs in right and left temporal regions.

#### 2.1.3. Mindfulness Meditation (MM)

Meditators have reduced physiological arousal and distraction from vague thoughts. It can be used for treating ADHD [19]. MM can induce neuroplasticity from earlier stages in self-referential processing and increased attention to internal and external stimulus. Self-referential processing is related to DMN (Default Mode Network) [20–24].

This study theorized various state and trait effects of MM on self-referential processing. Cahn et al. have measured the effect of Vipassana in terms of decreased delta and relative increase in theta over the frontal regions, though other usual changes over alpha, beta, and theta bands have not been observed. The main limitation of the study was unspecific effect of meditation practice on different frequencies, which should be well understood [24].

Ahani et al. [25] advanced the meditation research on MM. They supported the evidence of alpha and theta rise by observing the spectral analysis of recorded EEG. Although this randomized controlled trial was analyzed using the CG, the study was limited to novice meditators only [25]. Other MM techniques of IBMT (Integrative Body-Mind Training) and TBRT (Triarchic Body Pathway Relaxation) have been explored but still very little information is available and large scale considerations of these techniques are required for expert meditators [26, 27].

### 2.2. Transcending Meditation (TM)

Various physiological and neural effects in long-term Jacobson's Progressive Relaxation (PR) meditators (a classical method of relaxation), TM meditators, and group of beginners in PR were recorded [28]. Rare theta activity (5–7 Hz) was observed in all the three. Then, a detailed research on TM explained its effects that could be used for various clinical applications [29]. Comparing TM-Sidhi with TM program revealed higher frontal alpha1 and beta1 amplitudes and no change in coherence. Higher alpha1 and beta1 reflect automatic self-transcending and open monitoring, respectively. This analysis was conducted for frontal and parietal areas. For temporal amplitude averages, no significant changes were observed [30]. Limitation was no CG. It has also been made evident that TM could be used for stress reduction and improving the symptoms of ADHD (Attention Deficit Hyperactivity Disorder) [31, 32]. In a study, individual and group meditation effects were analyzed [33]. During individual meditation with closed eyes, strong delta coherence and theta coherence and no alpha coherence were observed. Distinct changes in alpha and theta coherence during group meditation show more relaxation reflected by subjects and less delta activity.

In another study, effects of TM on EEG alpha phase synchrony have been studied [34]. An increase in synchrony was observed around frontal and occipitoparietal lobes by a time domain method. It accounts for use of this style of meditation for better concentrated neural processes. Event related spectral perturbation (ERSP) analysis of EEG signal during TM practice has been presented for analyzing mind controllability of brain-computer interaction systems [35]. No CG was used for analysis.

### 2.3. Yoga Meditation

Evidences showed heightened theta EEG activity during Yoga Nidra and Sahaja Yoga but more control group designed study is needed [36, 37]. During Sahaja Yoga, LTM (Long-Term Meditators) showed higher theta and alpha1 power and STM (Short-Term Meditators) showed alpha2 desynchronization [38]. EEG mechanisms during Sahaja Samadhi (concentrative) meditation reflect increase in theta power and theta coherence in frontal area during deep meditation [39]. Again comparison with CG is required.

EEG pattern of a Kundalini Yoga master and Ananda Marga have been theorized as reflecting more alpha and theta band [40–44]. Still very few studies have been conducted for Yoga meditation considering the proficiency levels.

### 2.4. Other Meditation Practices

Paced Breathing (PB), which is a type of Su-Soku meditation, is a method of voluntary breathing [45]. It causes change in EEG parameters (increase in low frequency and high frequency alpha power and decrease in theta power). PB is advantageous compared to other meditative states [45].

An oscillatory mechanism of EEG during advanced breathing meditation, a new area to explore, has been studied in [46]. This finding supported the fact that the internalized attention is continuously enhanced and is noteworthy as reported by rise in theta activities, while relaxation is prominent only after deep phase of meditation as reported by changes in alpha activities [46].

### 2.5. Other Combined Studies

It has been revealed in a study that electric function connectivity differs in five traditions of meditation [47]. These are Tibetan Buddhists (TB), Qigong, Sahaja Yoga (SY), Ananda Marga Yoga (AY), and Zen [47]. The results have been taken for delta and beta2 bands because all five meditations showed significant changes in these two bands. In delta band of TB group, moving out of meditation showed left to right posterior connection while it showed anterior left to right posterior connectivity in AY group [47]. Such types of guesses evidently become impossible, for example, Qigong, since it includes a large number of connections. This research shows the common features in five traditions of meditation but with no CG and it did not explore the difference. Analysis of principal functional connectivities during delta band reflected that interdependence between different functions is lowered with practicing meditation irrespective of meditation style and inhibitory and excitatory (delta and beta2 band activities) brain region connectivities show this reduction [47, 48].

In another study, state effects of two meditation styles, mindfulness breathing and loving kindness (or metta) meditation, have been investigated [49]. Effects on prefrontal alpha asymmetry have been discussed. Subjects low in brooding showed response to loving kindness meditation while the opposite was observed for subjects with high brooding. Comparisons to rest group showed useful state effects of both the styles of meditation. It accounts for their clinical use for previously depressed patients, although various limitations like unspecific factors, small sample size, and novice subjects were observed in the study [49].

## 3. Controversial Studies

It has also been reported that meditation has an adverse effect of predisposing to epileptogenesis panic attacks, overexcited central nervous system, paradoxical rise in anxiety, becoming more hypercritical, disorientation, and high BP [10]. Regarding the use of meditation for high BP, more randomized clinical trials are required that could provide certain results [50]. Some positive effects might become negative if overexpressed in those with individual constitutional neurophysiological properties [EEG guided med.]. Deep meditation gives rise to high frequency gamma band bursts [51]. The patients with general epilepsy show increased gamma band activity especially 30–50 Hz in resting interictal EEG [52, 53]. Epileptiform EEG changes have been theorized in TM meditators. So some studies accounted for meditation resulting in epilepsy but some have rejected such claims [51–57]. Meditation predisposing to epilepsy is a controversial subject that requires scientific study.

During Bhramari Pranayama (BhPr), paroxysmal gamma (PGW) has been observed [58]. Using complex Morlet wavelets and Fourier analysis, features were extracted as high frequencies in beta (15–35) and gamma (>35) and increased theta activity. BhPr can represent epileptic activity since higher frequency epilepsy also exhibits such spiky shape and activity in temporal lobe as seen in BhPr [58]. Another study has brought light to TM as aggravating or treating epilepsy [59].

## 4. Signal Processing Challenges

Signal processing can add to this field of study [60]. The scalp electrodes, which contain the brain activity in terms of electric potentials, give signals which can be processed using SP algorithms to measure different mental activities and to reveal cognitive tasks. The internal language of the mind can be understood using different EEG patterns from electromagnetic field activity [61, 62]. EEG includes signals that are associated with awareness, encouragement, and cognitive load and affecting state of load [63–65].

EEG data is characterized by delta, theta, alpha, beta, and gamma [66]. A detailed description of assigned EEG bands has been given in Table 2.

A pronounced attempt to characterize EEG signatures for different types of meditation has been contributed by Travis and Shear [4]. They have not allocated rating but have valued the nature of meditation styles. A superior research in meditation is still required to categorize the EEG signatures corresponding to different meditation practices [4, 5].

Directly observing the nonlinear and nonstationary EEG raw data in time domain is a very tedious task [67]. So various linear and nonlinear signal processing techniques and their correlation to physiology have been proposed. Feature extraction algorithms acquire the spectral information from the preprocessed raw signal.

*Time frequency analysis* is beneficial in clarifying rhythmic information in EEG signals. Coherence techniques can also be used. Spectral covariance or coherence involves measurement of phase regularity between signal pairs in each frequency band. Higher alpha-theta coherence has been identified as meditation capability trait which appears intra- and interhemispherical in meditation [68]. As coherence cannot separate amplitude information and phase information while relating two signals, it measures roundabout phase locking only. Synchrony technique is being used rather than having spectral or coherence analysis. It is quantification of degree of phase locking between different narrowband signals [69].

*Linear methods* used are ICA (Independent Component Analysis), CSP (Common Spatial Patterns), LD (Linear Discriminator), and linear prediction method [70]. In other words, a wide range of algorithms extract the information in EEG signals like algorithms based on Fast Fourier Transform, Hilbert-Huang Transform, Wavelet Transform, rule based expert systems, and numerous other algorithms to define the temporal extents [70–72]. A method based on PBFT (Period Based Frequency Tracker) has been proposed to keep track of rhythmic variations of alpha frequencies [73]. Though STFT (Short Time Fourier Transform) can analyze such spectral variations, this method proved better in terms of fairer frequency resolution [73]. Also, problem of asymmetrical periodicity of EEG has been solved reliably and efficiently.

The inherent inhomogeneous characteristics and multinature are dealt with using* nonlinear signal processing techniques* on the basis of various parameters, for example, CD (Correlation Dimension), LLE (Largest Lyapunov Exponent), and H (Hurst exponent) [70]. Four-channel analysis of EEG has been given for Compressed Spectral Array (CSA), the running fractal dimension, and running attractor dimension [74]. CSA yields interesting features. The running attractor is more efficient in analyzing neural dynamics. Multiscale fractal dimension (MSFD) technique is another area of research to explain the multiscale temporal patterns of EEG [75]. Nonlinear methods are better than time domain, frequency domain, and linear methods.

## 5. Future Work and Challenges

A crisp and consistent observation of a particular EEG component and its changing direction as either increasing or decreasing for different meditation practices is still required. Numerous researches have been carried out to scientifically explore the underlying brain neuroelectrical effects during meditation but still neurophysiological effect of meditation is an open question. Though it has been theorized that a typical EEG signature of meditation can be increase in theta, alpha band power, decrease in at least alpha frequency, and spread of alpha coherence across cortex, its realization needs further research [4]. Also finding a comparable nonmeditating group as a control group is very difficult [11]. The main limitation is absence of control group. Whether the changes in EEG signals classify the restrained states of consciousness is questionable, although a pronounced clarification has been contributed by Travis and Shear. Further investigations are required to quantitatively represent the activities during FmTheta and activities concerning DMN during different styles of meditation. For EEG during DMN, the proposed approaches can be cross power spectra techniques, partial coherence function computation techniques, and operational architectonics techniques. Further studies have been suggested to consider extra frequency ranges and cross-correlation between EEG signals [75]. Very few conclusions have been derived regarding the expertise level of Yoga meditation. Neurobiological study of various types of Buddhist, Yoga, and other meditations styles is still to be explored. Also, meditation predisposing to epileptic seizure as well as some other negative effects is a controversial subject that requires technical discussion.

Signal processing can add to this field of study. EEG is the best diagnostic tool to provide firm information about brain activities and temporal dynamics related to these activities within millisecond range but if analyzing technique is poor it could result in misinterpretation and may ignore certain neural correlates [67]. So it can be said that EEG is misjudged highly. More processing tools and engineering approaches are required to be investigated for exploring EEG information [76].

Also meditation effects on the brain activity measured by EEG could be contaminated by the electromuscular artifacts. EEG rhythms show 6 times less power in 25–30 Hz band and 100 times less 40–100 Hz power in paralyzed subjects [77]. So muscle contamination is an important issue in defining gamma EEG during meditation.

## 6. Conclusion

Nowadays, complementary therapies like meditation are starting to be used in clinical practices. Understanding neuroelectrical effects of meditation is an important area of research, especially considering the application of meditation techniques in clinical practice and therapy. Studies based on EEG brainwave signals can help medical practitioners to check the activity level of brain and based on the health state, different meditation practices can be applied to progress mental fitness. In this paper, a broad spectrum of neural mechanics under a variety of meditation styles has been reviewed. A detailed quantitative analysis of various meditation states like Zen, CHAN, mindfulness, TM, Vipassana, Kundalini, Yoga, and other meditation styles has been described by means of EEG bands and coherence. It has been concluded that increased independent brain processes are observed compared to task-free resting during meditation. More rigorous studies are required with better design, considering client variables like personality characteristics to avoid risks, randomized controlled trials, long-term effects, and large sample sizes. More rigorous clinical trials that concentrate on the use of meditation are also required. Investigations on further activities can add neuroanatomy to explore the mechanisms of mind and body. Signal processing aspects in the field of cognitive neuroplasticity have also been discussed. A superior research is still required to categorize the EEG signatures corresponding to different practices.

In summary, highly developed approaches to the research of meditation will scientifically explore the underlying brain functions which may be beneficial for social well-being. A large scale research has been done to explore the neurophysiologic effects of meditation but still much is to be done to explore this area as suggested by various studies.

## Figures and Tables

**Table 1:** Meditation induced changes in EEG brainwaves.

Author	Meditation style	Subjects	Signal processing	Results
Sobolewski et al. [12]	Buddhist	26	—	↑ ERP^1^

Chang and Lo [13]	Zen	41	Wavelet	Alpha suppression (though more alpha than nonmeditators)

Huang and Lo [14]	Zen	23	—	↑ frontal alpha^2^ and occipital beta^3^

Kubota et al. [16]	So-Soku	25	Spectral analysis	↑ FmTheta (reflecting continuous attention)

Lo and Zhu [17]	CHAN	16	Wavelet decomposition and Mahalanobis fuzzy C-means	Time period of frontal alpha more than occipital alpha (reflecting calm mind)

Lo and Chang [18]	CHAN and Chakra focusing	20	Continuous Time Wavelet Transform	Higher lateral interaction of dominant alpha epochs in right and left temporal regions (reflecting more inward attention)

Berkovich-Ohana et al. [23]	MM	12	Spectral analysis	(i) State effect: ↑ prefrontal gamma (ii) Trait effect: ↓ gamma over frontal and midline areas

Cahn et al. [24]	MM	16	ICA	(i) ↓ frontal alpha (ii) ↑ frontal theta and gamma (reflecting better synchronized function)

Ahani et al. [25]	MMI	34	Spectral, phase analysis	(i) ↑ frontal theta (enhancement of attentional and working memory process) (ii) Minor ↑ in right temporal and occipital alpha (iii) ↑ beta and EEG synchrony^4^

Xue et al. [26]	IBMT	45	Network analysis	↑ FmTheta

Chan et al. [27]	TBRT	19	FFT	(i) ↑ alpha asymmetry index (measure of positive emotions) (ii) ↑ FmTheta

Warrenburg et al. [28]	PR and TM	6	—	Rare theta activity (5–7 Hz)

Travis [30]	TM and TM-Sidhi	26	Spectral analysis and coherence analysis	During TM-Sidhi (i) ↑ frontal alpha1 and beta1 (ii) No change in coherence

Newande and Reisman [33]	TM (individual and group study)	10	Time frequency analysis	*Individual study* (1) Open eyes (i) Strong coherence in delta and theta^5^ (ii) ↓ alpha coherence (2) Closed eyes slight delta, almost no theta, and significant alpha coherence (3) Experienced meditators reflect strong alpha coherence and shift to theta coherence
				*Group meditation* (1) Open eyes ↑ alpha being strongest, slight more delta and theta coherence than individual study (2) Closed eyes^6^ (i) ↑ alpha coherence and theta coherence (ii) ↓ delta activity

Hebert et al. [34]	TM	27	Time domain method	↑ alpha synchrony

Eskandari and Erfanian [35]	TM	10	Wavelet decomposition	(i) Alpha ERS and beta ERS during imagination of hand movement (ii) Alpha ERD during rest

Kjaer et al. [36]	Yoga Nidra meditation	8	Spectral analysis	(i) ↑ theta power (ii) ↓ alpha power

Aftanas and Golocheikine [37]	Sahaja Yoga	20	Nonlinear system theory: DCx	(i) ↑ theta1, theta2, and alpha1 powers (ii) ↓ beta3 (signifies problem solving and thinking)

Aftanas and Golocheikine [38]	Sahaja Yoga	58	FFT	(i) Long-Term Meditators: ↑ theta and alpha1 power (ii) Short-Term Meditators: alpha2 desynchronization

Baijal and Srinivasan [39]	Sahaja Samadhi	20	Spectral and coherence analysis	(i) ↑ frontal theta during deep meditation (ii) ↑ theta coherence (iii) ↓ theta in parietal

Arambula et al. [40]	Kundalini	1	Spectral analysis	↑ alpha on entering meditation

Elson et al. [44]	Ananda Marga	11	—	(i) ↑ theta (ii) Stable alpha-theta band

Ghista et al. [42]	Ananda Marga	4	—	↑ alpha

Khare and Nigam [43]	Ananda Marga	30	Spectral analysis	↑ alpha and beta

Y.-J. Park and Y.-B. Park [45]	PB	58	FFT	(i) ↑ alpha1 (ii) ↓ theta

Tsai et al. [46]	Advanced breathing	1	Single time series analysis	↑ bilateral alpha and theta

Lehmann et al. [47]	Tibetan Buddhists, QiGong, Sahaja Yoga, Ananda Marga, and Zen	13 15 14 14 15	Lagged intracortical coherence	Lowered interdependence between different functions as shown by delta and beta2 band activities

Barnhofer et al. [49]	Mindfulness breathing and loving kindness	8 7	FFT	Strong left prefrontal activation (reflects strong tendency for motivation and positivity)

Vialatte et al. [58]	Bhramari Pranayama	8	Complex Morlet wavelets and Fourier analysis	↑ frequencies in beta (15–35) and gamma (>35) and increased theta activity

1: stability towards negative stimuli, 2: more internalized attention, 3: open monitoring, 4: improved functional integration, 5: due to awake, alert, and open eyes, and 6: relaxed and deeper level of meditation.

**Table 2:** EEG brainwave frequency bands [4, 70, 78–81].

Frequency bands with subbands^*∗*^	Brain regions	Characteristics	Pathologies
Delta (<4 Hz)	(i) Frontal in adults (ii) Posterior in children	(i) Tendency to be of the highest amplitude (ii) During deep sleep stages in adults, appear in infants up to one year (iii) Slowest brain rhythms (iv) Minimal conscious brain involved (v) FIRDA (Frontal Intermittent Rhythmic Delta): 2 to 3 Hz having amplitude 50–100 mV generated frontally, in wake adults needing differentiation from artifacts like slow eye blinks (vi) OIRDA (Occipital Intermittent Rhythmic Delta): linked largely with seizures in children	(1) Focal delta activity: abnormal indicating lesions (2) Lower delta power: complex depression, continual migraine, closed posterior head/neck damage (3) Excess delta activity^*∗∗*^: learning disability, Alzheimer's disease, edema

Theta (4–8 Hz) (i) Theta1 (4–6 Hz) (ii) Theta2 (6–8 Hz)	Medial prefrontal and anterior cingulate cortex	(i) Amplitude 30–60 *µ*V (FmTheta) (ii) In normal children and infants, in adults during drowsiness, sleep, and meditation relaxed state (iii) Neural indicator of inner processes requiring self-management (iv) FmTheta: theta rhythm of 6-7 Hz centered around 6.5 Hz, a good indicator of continuous attention, linked with less anxiety, less phobic activity	In normal awaken adults, very small theta activity but a high value accounts for pathological conditions

Alpha (8–13 Hz) (i) Alpha1 (8–8.9 Hz) (ii) Alpha2 (9–10.9 Hz) (iii) Alpha3 (11–12.9 Hz)	(i) Posterior on each side of the head with higher amplitude on leading side (ii) Faster at posterior, slower at anterior recording positions	(i) Amplitude < 50 *μ*V (ii) Dominant frequency in adults (iii) Tasks requiring attention, semantic memory (iv) Easily seen with closed eyes and under mental inactive conditions (v) Lower alpha band (alpha1, alpha2) is an index of internalized attention (vi) High alpha band (alpha3) is an index of engagement in task demands (vii) ERD: during bigger task demands, alpha is desynchronized and theta is synchronized (viii) Alpha blocking occurs when eyes are opened in a well lit room. The waves are dominated by fast low amplitude beta rhythms (ix) Mu rhythm: alpha activity in sensorimotor cortex	Alpha coma, a diffused alpha in EEG occurs in coma which does not respond to external stimuli

Beta (13–30 Hz) (i) Beta1 (13–18 Hz) (ii) Beta2 (18–22 Hz) (iii) Beta3 (22–30 Hz)	(i) Frontocentral (ii) Posterior-occipital in infants which shifts to frontal areas as we grow up	(i) Usually amplitude ≤ 30 *μ*V (ii) In all age groups in alert and anxious subjects enhanced by expectation states (iii) Beta1 consists of corticothalamic feedback loop that changes attention level, beta bursts shift the system into attention state that allow gamma synchronization (iv) Beta2 during focused managerial processing	(1) Decreased beta activity: focal lesions, stroke, or tumor diffused encephalopathy such as anoxia (2) Excess beta activity during alcoholism along with decreased alpha and theta

Gamma (>30 Hz)	Different brain regions	(i) Extremely fast frequency with lowest amplitude (ii) Provides synaptic plasticity for long-term memory by closely tracking the local change in blood flow (iii) Higher in focused stimuli	Sometimes of no clinical interest and filtered out in EEG recordings

^*∗*^Although most researchers use these universally accepted frequency ranges, since frequency varies with age, neurological diseases, brain volume, task requirements, and memory performance, some researchers use their own range of boundaries.

Also, in some studies, decimal values have been used for defining frequency bands instead of whole numbers.

^*∗∗*^Increased delta activity considered with normal adults performing calculations, reaction time tests in some studies.
